# Syrians' awareness of cardiovascular disease risk factors and warning indicators: a descriptive cross-sectional study

**DOI:** 10.1038/s41598-023-32026-4

**Published:** 2023-04-25

**Authors:** Sarya Swed, Hidar Alibrahim, Haidara Bohsas, Wael Hafez, Mohammed Amir Rais, Sheikh Shoib, Ebraheem Albazee, Mohamed E. G. Elsayed, Bisher Sawaf, Amr Farwati, Mohammed Najdat Seijari, Naim Battikh, Nour Shaheen, Nafeth Ibrahem, Ahmad Alsaleh, Ka Yiu Lee, Amine Rakab

**Affiliations:** 1 Faculty of Medicine, Aleppo University, Aleppo, Syria; 2 NMC Royal Hospital, 16th Street, Khalifa City, Abu Dhabi, UAE; 3 Medical Research Division, Department of Internal Medicine, The National Research Centre, Cairo, Egypt; 4 Faculty of Medicine of Algiers, University of Algiers, Algiers, Algeria; 5 JLNM Hospital, Rainawari, Srinagar, India; 6 Directorate of Health Services, Srinagar, J&K India; 7 Kuwait Institute for Medical Specializations, Kuwait City, Kuwait; 8 Department of Psychiatry and Psychotherapy III, University of Ulm, Leimgrubenweg 12-14, 89075 Ulm, Germany; 9 Department of Psychiatry, School of Medicine and Health Sciences, Carl von Ossietzky University Oldenburg, Oldenburg, Germany; 10 Department of Internal Medicine, Hamad Medical Corporation, Doha, Qatar; 11 John H. Stroger, Jr. Hospital of Cook County, Chicago, USA; 12 Faculty of Medicine, Alexandria University, Alexandria, Egypt; 13 Faculty of Medicine, Damascus University, Damascus, Syria; 14 Department of Health Sciences, Mid Sweden University, Sundsvall, Sweden; 15 Weill Cornell Medical College, Doha, Qatar

**Keywords:** Diseases, Risk factors

## Abstract

The awareness of cardiovascular diseases (CVDs) contributes to the complications and fatality rates from these diseases among individuals; however, no previous study in Syria was conducted on this topic; thus, this study aims to assess Syrians' awareness of CVDs warning symptoms and risk factors. This online cross-sectional study was performed in Syria between the 1st and 25th of August 2022. The inclusion criteria for the sample were citizens of Syria over 18 who currently reside in Syria. The questionnaire included open- and closed-ended questions to assess the awareness of CVDs. A total of 1201 participants enrolled in the study with a response rate of 97.2%; more than half of the participants (61.4%) were aged 18–24. The most recognizable risk factors and warning signs when asking close-ended and open-ended questions were smoking (95.2%, 37.1%) and chest pain (87.8%, 24.8%), respectively. Overall knowledge scores for risk factors and warning signs were (61.5%). Regarding knowledge score of CVDs risk factors and warning signs, participants aged 45–54 scored higher than other age groups, and respondents with a university education level had a higher score than other educational levels (15.7 ± 0.3), (14.5 ± 0.1), respectively. Participants aged 45–54 have a higher probability of good knowledge of CVDs risk factors and warning signs than participants aged 18–24 (OR = 4.8, *P* value < 0.001), while participants living in the countryside were less likely to have good knowledge of CVDs risk factors and warning signs than city residents (OR = 0.6, *P* value < 0.05). According to our results, there is inadequate knowledge of the risk factors and warning signs of CVDs. Consequently, there is a greater need to raise CVD awareness and learning initiatives on the disease's risk factors and symptoms.

## Introduction

Most fatalities worldwide are caused by cardiovascular diseases (CVDs), which takes part in non-communicable disease (NCDs)^1^. The global burden of cardiovascular disease (CVD) is a significant concern due to its substantial health impact, which is predicted to increase^1^. NCDs cause most mortality worldwide, and cardiovascular disorders account for over half of them^2^. The main aetiology of CVDs is damaging the heart and blood vessels^1^ which causes ischemic heart disease, stroke, heart failure, peripheral arterial disease, and other cardiac and vascular conditions^1^. Unhealthy lifestyle habits, including unhealthy nutrition, lack of exercise, cigarette use, and excessive alcohol use, are major contributors to cardiovascular disease and stroke development. CVDs caused 17,800,000 deaths worldwide in 2017, accounting for 330,000,000 years of life lost and an additional 35,600,000 years of incapacity^2,3^. In 2030, it is anticipated that ischemic heart disease (IHD) and cerebrovascular disease (stroke), will be the leading killers worldwide^2^. Those suffering from CVDs and other non-communicable disorders in underdeveloped nations have less access to effective medical care that suits their requirements. In addition, compared to high-income countries (HICs), low and middle-income countries (LAMICs) had a much higher prevalence of CVD among adults of productive age^4^. Inadequate knowledge of CVDs risk factors was found as a remarkable obstacle to the treatment and prevention process, as well it affects people's attitudes towards these diseases^5^. Focusing on the risk factors and underlying causes is necessary to combat the CVD prevalence in developing countries. Conversely, several studies have shown that knowledge and awareness play a role in healthy behaviours^6,7^, which affects patients' behaviour and attitude, improves adherence to therapy, and reduces complications possibility^8^. These models highlight the significance of evaluating people's beliefs, opinions, and attitudes to understand observed behaviours and promote behavioural change, even when they vary in substance and viewpoint. Syria’s CVD burden is due mainly to rising trends in risk factors, including smoking, obesity, blood pressure, cholesterol, and diabetes^9^. Before the crisis began in 2011, NCDs (mostly CVDs, cancer, and diabetes) were responsible for 77% of all fatalities in the country^10^. Previous CVD management and prevention advances in Syria were not based on epidemiological data or rigorous population studies but recycled foreign information and implemented it regardless of applicability^11^. No studies have been conducted that measure the awareness of CVDs and associated risk factors in the general Syrian population. This research aims to assess the people’s knowledge of CVD risk factors and warning indicators in Syria and further investigate knowledge-related factors.

## Methods

### Study design and setting

This cross-sectional study was conducted in Syria between August 1 and 25, 2022, to assess Public knowledge of risk factors and warning signs for cardiovascular disease among Syrian adults. Participants included the Syrian population above 18 and who live in Syria, whereas we excluded Syrian people less than 18 years, who live outside of Syria, and who didn't complete the survey. The aims of the study, the name of the research team, the participant's ability to withdraw from the study, their right to privacy and data protection, and the fact that only data that was fully recorded would be examined were all given to all participants at the first page of the google form survey. The questionnaire was developed based on previous research conducted in Tanzania^12^. It was translated from English to Arabic by language experts who were healthcare professionals and had great expertise in biomedical research. We double-checked its correctness to ensure it was appropriate for the Syrian language; however, the utilized questionnaire was uploaded as supplementary material (Table S1 Table 1). Specific collaborators from many medical Syrian colleges (Data Collection Group) collected the data by disrupting the online survey, which was generated at the google form website, on social media platforms such as WhatsApp, Telegram, and Facebook. Also, the data collection group was mentored by a supervisor who has access to the google form website. Convenience and snowball tactics were used to get data from respondents.

**Table 1:** Demographic characteristics of study participants.

Variable	n (%)	Variable	n (%)
Age group		Height	
18–24	738 (61.5)	150>	14 (1.17)
25–34	185 (15.4)	150–159	297 (24.7)
35–44	114 (9.5)	160–169	425 (35.39)
45–54	99 (8.2)	170–179	330 (27.48)
55–64	65 (5.4)	180–189	128 (10.66)
Gender		190≤	7 (0.6)
Male	728 (60.6)	Weight	
Female	473 (39.4)		
Place of residency		50<	128 (10.7)
City	801 (73.4)	50–59	279 (23.2)
Countryside	320 (26.6)	60–69	303 (25.2)
Level of education			
No formal education	23 (1.9)	70–79	595 (21.8)
Primary school	11 (0.9)	80–89	139 (11.6)
Middle school	42 (3.5)	90≤	90 (7.5)
Secondary school	145 (12.1)	Smoking	
University (Bachelor's or Master's)	980 (81.6)	No	898 (74.8)
		Yes	303 (25.2)
Marital status		Alcohol consumption	
Single	841 (70)	No	1115 (92.8)
Married	324 (27)	Yes	86 (7.2)
		Family history of diabetes	
Divorced	20 (1.7)	No	1144 (95.3)
Widowed	16 (1.3)	Yes	57 (4.7)
Monthly income		Hypertensive	
High	38 (3.2)	No	1077 (89.7)
Good	390 (32.4)	Yes	124 (10.3)
Middle	670 (55.8)	Family history of heart disease	
Low	103 (8.6)	No	740 (61.6)
		Yes	461 (38.4)
Occupation		Family history of hypertensive	
Student	567 (47.2)	No	483 (40.2)
Farmer	17 (1.42)	Yes	748 (59.8)
		Family history of diabetes	
House wife	81 (6.74)	No	656 (54.6)
Employed	179 (14.9)	Yes	545 (45.4)
		Concern of heart disease	
Petty business	65 (5.4)	No	674 (56.1)
Medical sector	193 (16.1)	Yes	527 (43.9)
		Concern of hypertensive	
Other	99 (8.24)	No	661 (55)
Concern of diabetes			
No	651 (54.2)	Yes	540 (45)
Yes	550 (45.8)		

Sample size calculation.

The sample size was calculated by performing the following formula^13^:

$${\text{Sample size}} = {\text{ Z}}_{{{1} - {\text{a}}/{2}}}^{2} {\text{P}}\left( {{1} - {\text{P}}} \right)/{\text{d}}^{{2}} ;{ 1}.{96}^{{2}} 0.{16 }\left( {{1} - 0.{16}} \right)/0.0{5}^{{2}} = {2}0{4}$$

Z1 − a/2 = In our study P value is considered significant under 0.05 so Z1 − a/2, which equals 1.96.

P = Predicted proportion in Tanzania population who have adequate level of knowledge toward CVDs; 16.3%d = Absolute precision; 0.05

### Measures

#### Sociodemographic variables and work-related characteristics

Age, gender, marital status, education level, and employment were all socio-demographic factors. The level of education was determined by asking about the highest degree of education attained: none, primary education, middle school secondary education, and college/university. At the time of data collection, marital status was grouped into four groups, single, married, divorced, and widowed and separated. The evaluated occupations were classified as student farmers, homemakers, employed (public/private), small business owners, and others. We also asked if the participant belonged to the healthcare sector. The economic status of the participants was assessed by asking about Monthly income: bad, moderate, good, and very good (income per capita).

#### Knowledge toward CVD risk factors and warning signs

We depended on the cutoff points of the utilized questionnaire from the reference study. Open-ended questions were used to evaluate participants' knowledge of risk factors and warning signs, followed by closed-ended questions requiring participants to choose risk factors and warning signs from a list of “yes” or “no” questions. Each accurate answer received a knowledge score of 1 (one), while incorrect responses received a 0 (zero). Closed-ended questions were used to determine knowledge scores. The overall score for knowledge of risk factors and warning signs was then calculated by adding the scores for each category. The top overall knowledge score was 19 points. Participants were categorized as having “good knowledge” if their total knowledge score was greater than 14, “moderate knowledge” if their total knowledge score was between 8 and 13, “poor knowledge” if their total knowledge score was between 1 and 7, and “not knowledgeable” if they received no score.

##### Knowledge toward CVD risk factors

The questionnaire included 10 questions regarding risk factor knowledge. Open-ended questions (participants were asked to mention risk factors spontaneously) were used to test risk factor knowledge, followed by closed-ended questions (participants were asked to identify risk factors from a set of “yes/no” questions). Overall knowledge scores for risk factors were divided into four categories: high knowledge (7–10 points), moderate knowledge (4–6 points), weak knowledge (1–3 points), and no knowledge at all (0 point).

##### Knowledge toward CVD warning signs

The awareness of warning indicators section of the questionnaire has nine items. After open-ended questions in which participants were requested to name warning indicators spontaneously, knowledge of warning signs was evaluated using closed-ended questions in which participants were asked to choose warning signs from a list of “yes/no” questions. The total scores for knowledge warning signals were divided into four categories: excellent (7–9 points), moderate (4–6 points), weak knowledge (1–3 points), and not at all knowledgeable (0 point).

#### Medical history and behavioral risk factors

Participants were queried if they had ever been diagnosed with either high blood pressure or diabetes mellitus. Using a modified WHO STEPs questionnaire^12^, lifestyle-related CVD risk factors such as drinking alcohol (Consume alcohol or do not consume at all), smoking, and dietary patterns were evaluated.

### Pilot study

We sent the online survey to 50 Syrian individuals to ensure that the inquired questionnaire was clear and readable for the respondents. Then we modified the questionnaire depending on the suggested feedback. Although we took the questionnaire from the published study conducted in another country (Tanzania), we again confirmed its reliability and validity by computing Cronbach's alpha for each performed scale (Knowledge of risk factors and warning signs of CVD).

### Ethical consideration

The IRB was received from the Ethical Society for Scientific Research in Syria (IRB = SA764). All methods were performed in accordance with the relevant guidelines and regulations. Informed consent was obtained from all participants. Participants were given a URL to access the online survey on Google, and the first page of the survey questioned participants whether they agreed to complete the questionnaire. Before completing the questionnaire, they are sent to the following page, which contains comprehensive information on the research. Complete the questionnaire in between 5 and 12 min. All responses were saved in a protected online database.

### Statistical analysis

After the data collection was finished, the data were exported to SPSS and stored in a secure file on Excel. Software for Windows running IBM's Statistical Package for Social Sciences (SPSS Inc., Chicago, IL, USA) version 28 was used to input and analyze the data. The means and standard deviations of continuous variables were summarized using descriptive statistics and the frequencies and proportions of categorical variables. Independent samples t-test and ANOVA tests were performed to detect the statistical difference between the categorical and continuous variables (score of each used scale). Shapiro–Wilk test was performed to assess the data distribution, and we made sure that all continuous variables had normal distribution; then, they were presented as mean and standard deviation. To perform binary logistic regression and prediction, good knowledge of CVDs, knowledge of CVD risk factors and warning signs was first dichotomized as follows: adequate knowledge of risk factors was considered as score ≥ 7/10, adequate knowledge of warning signs of CVDs was considered as score ≥ 7/9, in addition, the adequate knowledge of the combing two scales was considered as overall score ≥ 14/19. Binary logistic regression was performed to determine the relationship of each independent variable (sociodemographic variables) to predict the existence of adequate knowledge toward CVD (dependent variable), with adjusting for all co-variates (independent variable). The overall fitness of the model was assessed using the Pearson Chi-square test and Hosmer–Lemeshow goodness-of-fit test. Adjusted odds ratio (AOR) with their corresponding 95% confidence intervals (95% CI) are presented as measures of association. Statistical significance was accepted based on a two-sided *P* value ≤ 0.05.

### Ethics approval and consent to participate

All experimental protocols were approved by the Ethical Society for Scientific Research in Syria.

## Results

### Characteristics of study participants

The questionnaire was sent to 1235 participants; 15 refused to complete the survey, and 19 did not meet the inclusion criteria; thus, the final sample size was 1201. Participants aged 18–24 were (61.4%) of the study sample, and only (5.4%) were aged 55–64. Males compress more than half of the study participants 60.6%, and the majority of participants live in the city (73.4%); however, most of the participants have a Bachelor's or Master's degree of educational level (81.6%), and only (19%) do not have formal education., there were (25.2%) smokers among the participant, and only (7.2%) were alcohol consumers. Only (4.7%) of participants had a diabetes history, and (10.3%) had hypertension. Family history of diabetes was found in (45.4%) of the participants, and (59.8%) have a family history of hypertension (Table 1).

### Knowledge of risk factors and warning signs

#### Open-ended questions

When we asked the participants open-ended questions, the most recognizable CVD risk factors were smoking (37.1%), obesity (29%), and cholesterol (25.6%). Regarding the warning signs, chest pain (24.8%), shortnesss of breath (21.1%), and severe headache (15.3%) were the most mentioned warning signs. Participants correctly identified 5–10 risk factors and 5–9 warning signs, 24.2% and 12.3%, respectively.

#### Close-ended questions

By asking the respondents close-ended questions, most of them defined smoking (95.2%), obesity (93.6%), and cholesterol (91%) as risk factors for CVDs. However, the majority of participants identified chest pain (87.8%), shortness of breath (78.9%), and pain in the arm or shoulder (76.2%) as warning signs for CVDs. Participants correctly recognized 5–10 risk factors and 5–9 warning signs, 94.9% and 72.7%, respectively (Table 2).

**Table 2:** Knowledge of risk factors and warning signs.

Risk factors		Warning signs	
Variable	n (%)	Variable	n (%)
Open-ended questions			
The risk factors from the participants for CVD		The warning signs from the participants for CVD	
Old age	172 (14.3)	Severe headache	184 (15.3)
Obesity	348 (29)	Chest pain	298 (24.8)
Hypertension	174 (14.5)	Shortness of breath	253 (21.1)
Diabetes mellitus	161 (13.4)	Sweating	136 (11.3)
Cholesterol	307 (25.6)	Vomiting	84 (7.0)
Smoking	445 (37.1)	Pain in the jaw or neck	6 (0.5)
Alcohol use	205 (17.1)	Pain in the arms or shoulder	122 (10.2)
Physical inactivity	99 (8.2)	Loss of consciousness	68 (5.7)
Family history of stroke	177 (14.7)	Dizziness	91 (7.6)
Stress	181 (15.1)	Number of warning signsidentified	
Number of risk factors identified			
0	478 (39.8)	0	680 (56.6)
1–4	432 (36)	1–4	373 (31.1)
5–10	291 (24.2)	5–9	148 (12.3)
Close-ended questions			
Selecting risk factors for CVDs		Selecting warning signs	
Old age	1007 (83.8)	Severe headache	617 (51.4)
Obesity	1124 (93.6)	Chest pain	1055 (87.8)
Hypertension	1085 (90.3)	Shortness of breath	947 (78.9)
Diabetes mellitus	816 (67.9)	Sweating	823 (68.5)
Cholesterol	1093 (91)	Vomiting	456 (38)
Smoking	1143 (95.2)	Pain in the jaw or neck	391 (32.6)
Alcohol use	978 (81.4)	Pain in the arms or shoulder	915 (76.2)
Physical inactivity	959 (79.9)	Loss of consciousness	882 (73.4)
Family history of stroke	925 (77)	Dizziness	897 (74.7)
Stress	1035 (86.2)	Number of warning signs	
Number of risk factors identified			
0	9 (0.7)	0	32 (2.7)
1–4	28 (2.3)	1–4	296 (24.6)
5–10	1164 (96.9%)	5–9	873 (72.7)

### Overall knowledge of CVDs risk factors an warning signs

More than half of the respondents have shown good knowledge of CVDs risk factors and warning signs (61.5%), while only 3.4% have shown poor knowledge in Table 3.

**Table 3:** Participants’ knowledge scores for risk factors, warning signs:

Variable	n (%)
Knowledge scores on risk factors for CVD	
Adequate or Good knowledge (7–10 points)	1033 (86.1)
Moderate knowledge (4–6 points)	149 (12.4)
Poor knowledge (1–3 points)	10 (0.8)
Not knowledgeable (0 point)	9 (0.7)
Knowledge scores on warning signs for CVD	
Adequate or Good knowledge (7–9 points)	496 (41.3)
Moderate knowledge (4–6 points)	514 (42.8)
Poor knowledge (1–3 points)	159 (13.2)
Not knowledgeable (0 point)	32 (2.7)
Overall knowledge scores for risk factors and warning signs	
Adequate or Good knowledge (> 14 points)	739 (61.5)
Moderate knowledge (8–13 points)	416 (34.6)
Poor knowledge (1–7 points)	41 (3.4)
Not knowledgeable (0 point)	5 (0.4)

### Association of socio-demographic factors and knowledge score

Regarding CVDs risk factors and warning signs knowledge score, participants aged 45–54 scored higher than other age groups (Mean ± SD; 15.75 ± 0.31, *P* value < 0.05), as well participants living in the city have a greater score than countryside residents (14.41 ± 0.12, *P* value < 0.05). Respondents with a University education level scored higher than other educational levels (14.54 ± 0.11, *P* value < 0.05) as shown in Table 4.

**Table 4:** The Difference between the sub-groups of knowledge toward risk factors, and warning signs of CVDs among the study sample.

Variable	Categories	Risk factors			Warning signs			Risk factors and warning signs		
		Mean (SD) 8.47 (0.077)	95%CI (lower–upper)	*P* value	Mean (SD) 5.81 (0.097)	95%CI (lower–upper)	*P* value	Mean (SD) 14.28 (0.153)	95%CI (lower–upper)	*P* value
Age group				< 0.001			< 0.001			< 0.001
	18–24	8.35 (0.07)	8.22–8.49		5.7 (0.083)	5.53–5.86		14.05 (0.136)	13.78–14.32	
	25–34	8.36 (0.138)	8.09–8.63		5.55 (0.180)	5.2–5.91		13.91 (0.271)	13.38–14.45	
	35–44	8.49 (0.148)	8.2–8.78		5.87 (0.213)	5.45–6.29		14.36 (0.299)	13.77–14.95	
	45–54	9 (0.166)	8.67–9.33		6.75 (0.202)	6.35–7.15		15.75 (0.314)	15.12–16.37	
	55–64	9.15 (0.16)	8.83–9.47		6.38 (0.258)	5.87–6.9		15.54 (0.356)	14.83–16.25	
Gender				0.071			0.445			0.205
	Female	8.44 (0.064)	8.31–8.56		5.82 (0.078)	5.66–5.97		14.25 (0.125)	14.01–14.5	
	Male	8.51 (0.091)	8.33–8.68		5.81 (0.116)	5.58–6.04		14.32 (0.181)	13.96–14.67	
Place of residency				0.183			0.011			0.016
	City	8.51 (0.061)	8.39–8.63		5.9 (0.077)	5.75–6.05		14.41 (0.121)	14.17–14.65	
	Countryside	8.34 (0.106)	8.13–8.55		5.57 (0.129)	5.32–5.83		13.91 (0.202)	13.51–14.31	
Level of education				< 0.001			0.013			< 0.001
	No formal education	8.61 (0.371)	7.84–9.38		5.83 (0.53)	4.71–6.94		14.43 (0.659)	13.07–15.8	
	Primary school	6.55 (0.743)	4.89–8.2		5 (0.831)	3.15–6.85		11.55 (1.442)	8.33–14.76	
	Middle school	7.71 (0.377)	6.95–8.48		5.12 (0.409)	4.29–5.95		12.83 (0.639)	11.54–14.12	
	Secondary school	7.66 (0.165)	7.34–7.99		5.42 (0.166)	5.09–5.75		13.08 (0.284)	12.52–13.64	
	University (Bachelor's or Master's)	8.63 (0.055)	8.52–8.74		5.91 (0.73)	5.77–6.05		14.54 (0.113)	14.32–14.77	
Marital status				0.095			0.050			0.026
	Single	8.42 (0.063)	8.29–8.54		5.69 (0.078)	5.53–5.84		14.1 (0.124)	13.86–13.35	
	Married	8.52 (0.103)	8.32–8.73		6.1 (0.128)	5.85–6.35		14.62 (0.202)	14.23–15.02	
	Divorced	8.85 (0.335)	8.15–9.55		6.7 (0.524)	5.6–7.8		15.55 (0.709)	14.07–17.03	
	Widowed	9.13 (0.427)	8.2–10.04		5.69 (4.33)	4.33–7.05		14.81 (0.853)	13.0–16.63	
Monthly income				0.089			0.900			0.521
	High	8.61 (0.378)	7.84–9.37		5.95 (0.468)	5–6.9		14.55 (0.744)	13.05–16.06	
	Good	8.55 (0.09)	8.37–8.72		5.85 (0.111)	5.64–6.07		14.4 (0.177)	14.05–14.75	
	Middle	8.39 (0.07)	8.26–8.53		5.8 (0.087)	5.63–5.97		14.19 (0.137)	13.92–14.46	
	Low	8.55 (0.19)	8.18–8.93		5.72 (0.259)	5.21–6.23		14.27 (0.385)	13.51–15.04	
Occupation				< 0.001			< 0.001			< 0.001
	Student	8.36 (0.077)	8.21–8.52		5.58 (0.094)	5.4–5.77		13.95 (0.151)	13.65–14.24	
	Farmer	8.41 (0.429)	7.5–9.32		5.65 (0.717)	4.13–7.17		14.06 (0.941)	12.06–16.05	
	House wife	7.69 (0.238)	7.22–8		5.44 (0.225)	5–5.89		13.14 (0.416)	12.31–13.96	
	Employed	8.51 (0.134)	8.25–8.78		5.78 (0.175)	5.43–6.12		14.29 (0.262)	13.77–14.81	
	Petty business	8.98 (0.2)	8.58–9.38		6.35 (0.315)	5.72–4.98		15.34 (0.448)	14.44–16.23	
	Medical sector	9.05 (0.11)	8.84–9.27		6.7 (0.145)	6.42–6.99		15.76 (0.224)	15.31–16.2	
	Other	8.1 (0.188)	7.73–84.7		5.44 (0.245)	4.96–5.93		13.55 (0.364)	12.82–14.27	

### Predictors of adequate knowledge of risk factors and warning signs

A binary logistic regression was employed to determine the appropriate level of understanding regarding CVD risk factors and warning signs. A higher chance of good knowledge about CVDs warning signs was observed by individuals aged 45–54 than respondents aged 18–24 (OR = 1.91, *P* value < 0.05); participants aged 45–54 were more likely to have good knowledge of CVDs risk factors and warning signs than respondents aged 18–24 (OR = 4.81, *P* value < 0.001), while participants living in the countryside have a lower probability of good knowledge about CVDs risk factors and warning signs than city residents (OR = 0.67, *P* value < 0.05), as shown in Table 5.

**Table 5:** Predictors of adequate knowledge of risk factors and warning signs.

Variable	Knowledge of risk factors		Knowledge of warning signs		Knowledge of risk factors and warning signs	
	AOR* (95%CI)	*P* value	AOR* (95%CI)	*P* value	AOR* (95%CI)	*P* value
Age group						
18–24	Ref					
25–34	0.098 (0.028–0.346)	< 0.001	0.844 (0.555–1.284)	0.428	1.043 (0.677–1.606)	0.848
35–44	0.193 (0.058–0.639)	0.007	1.231 (0.68–2.227)	0.492	1.933 (1.041–3.589)	0.037
45–54	0.507 (0.147–1.744)	0.281	1.918 (1.014–3.627)	0.045	4.814 (2.337–9.915)	< 0.001
55–64	0.604 (0.166–2.193)	0.443	1.809 (0.885–3.696)	0.104	3.544 (1.584–7.931)	0.002
Gender						
Female	Ref					
Male	1.443 (0.984–2.116)	0.061	1.003 (0.771–1.304)	0.104	0.896 (0.682–1.178)	0.432
Place of residency						
City	Ref					
Countryside	1.122 (0.76–1.657)	0.561	0.783 (0.59–1038)	0.089	0.676 (0.51–0.898)	0.007
Level of education						
No formal education	Ref					
Primary school	0.84 (0.204–3.456)	0.506	0.225 (0.038–1.335)	0.101	0.104 (0.016–0.658)	0.016
Middle school	0.132 (0.034–0.515)	0.004	0.424 (0.137–1.313)	0.137	0.616 (0.2–1.902)	0.400
Secondary school	0.39 (0.169–0.899)	0.027	0.652 (0.245–1.737)	0.392	0.608 (0.221–1.675)	0.336
University (Bachelor's or Master's)	0.364 (0.23–0.574)	< 0.001	0.936 (0.365–2.395)	0.889	1.441 (0.54–3.843)	0.465
Marital status						
Single	Ref					
Married	0.48 (0.053–4.359)	0.514	1.623 (1.026–2.568)	0.039	1.164 (0.722–1.877)	0.533
Divorced	0.327 (0.038–2.778)	0.306	1.596 (0.564–4.518)	0.378	2.724 (0.747–9.926)	0.129
Widowed	0.453 (0.031–6.57)		1.924 (0.613–6.039)	0.262	1.151 (0.338–3.92)	0.822
Monthly income						
High	Ref					
Good	0.948 (0.306–2.938)	0.927	0.806 (0.387–1.679)	0.565	1.128 (0.514–2.475)	0.763
Middle	1.093 (0.561–2.128)	0.785	0.769 (0.375–1.58)	0.475	1.020 (0.472–2.205)	0.959
Low	0.916 (0.556–1.922)	0.916	0.983 (0.433–2.23)	0.967	1.522 (0.635–3.649)	0.346
Occupation						
Student	Ref					
Farmer	2.351 (1.254–4.408)	0.008	1.465 (0.5–4.297)	0.486	1.009 (0.33–3.088)	0.988
House wife	1.733 (0.4–7.503)	0.462	0.506 (0.257–0.995)	0.048	0.44 (0.225–0.862)	0.017
Employed	0.602 (0.256–1.416)	0.245	0.775 (0.48–1.251)	0.297	0.576 (0.354–0.936)	0.026
Business	1.110 (0.542–2.271)	0.776	1.421 (0.73–2.767)	0.301	1.064 (0.503–2.251)	0.872
Medical sector	2.971 (0.879–10.047)	0.08	2.192 (1.542–3.116)	< 0.001	2.467 (1.646–3.697)	< 0.001
Other	4.372 (1.932–9.896)	< 0.001	0.667 (0.393–1.132)	0.133	0.634 (0.379–1.06)	0.082

*AOR: Adjusted odds ratio, which the performed logistic regression model included all co-variates.

## Discussion

Cardiovascular diseases affect the heart and all the blood arteries in the body. It is a significant issue affecting public health all around the globe^14,15^. Results from our research demonstrated that most of the population, or 93%, is already familiar with cardiovascular diseases (CVDs), with family, neighbours, and healthcare professionals being the primary sources of their CVDs knowledge which is consistent with results from a Saudi study in Riyadh city conducted by Ahmed et al.^16^ and findings reported by a study conducted in Ghana by Olutobi et al.^16^. These findings are also consistent with research on the Nigerian population^17,18^ and the Tanzanian population^12^. Our findings showed that participants have great knowledge of close-ended questions because open questions need a higher level of memory than closed-end questions to remember the warning signs and the risk factors of cardiovascular diseases. Almost participants (99.3%) identified at least one CVDs risk factor in contrast to findings reported from a study conducted in Kuwait^19^ where only 40% of the study population had sufficient information about risk factors. In our study, at the top were chosen cigarettes (95.2%), similarly found in results from a cross-sectional study among Filipino seafarers (68.4%) conducted by Ernesto et al.^20^. However, it is important to mention that knowledge through open-ended questions was poor, with (39.8%) of participants that couldn't mention any risk factors. Additionally, concerning warning signs, knowledge through closed-ended questions (97.3%) was higher than open-ended questions (43.4%). For CVDs knowledge risk and alarming factors scores, findings showed that 793 (61.5%) participants had good knowledge, and only 46 (3.8%) had poor or no knowledge of CVDs risk and alarming factors. The current study provides significant findings since results are strongly concomitant with several recent studies. Chest pain was the most incriminated symptom, which was the same found in a study conducted in Pakistan by Jafary et al.^21^. Furthermore, old age being the most identified risk factor in our study was also found to be the most chosen risk factor in a Tanzanian Study^12^. Also, CVDs knowledge, despite being satisfactory in our study, is found to be much higher in specific populations such as healthcare workers^2^ and among university staff ^22^ in two studies conducted in Nigeria. Additionally, we discovered in our research that several subgroups had a better level of awareness about the risk factors and warning signs of cardiovascular diseases (CVDs) than others. Our study results show that people aged 45–54 have a higher chance of good knowledge of CVDs risk factors and symptoms; this result was similar to a study conducted in Iran which shows that people above 40 have better knowledge of CVDs^23^. Interestingly, a study conducted in Kuwait shows that people aged 50–59 have a greater knowledge of CVDs than other age groups^19^; however, a chines study reported that the age group between 50 and 64 have a higher awareness of CVDs than older or younger age groups^24^. The increased knowledge among older age groups could be explained by the fact that older people seek about their health more than younger people since they face more health issues in old age and more chronic diseases occur with them.

This includes those who work in the medical field because they are in close touch with patients who have CVDs, as well as undergraduate medical students who studied this illness portion at each basic and clinical stage of their education. In addition, people who live in cities have a higher level of medical knowledge than those who live in rural areas. This is because medical education is more prevalent in urban areas. The knowledge of cardiovascular diseases in Syria is satisfactory and well in the closed questions; however, the knowledge level in the open questions was poor and inadequate. Therefore, it is possible to detect a significant degree of concern among Syrians about their lack of information about CVDs. This is because Syria's educational and health care levels have been worsened following 11 years of civil war, which destroyed many hospitals and physicians' immigration to neighbouring countries to get enough resources for survival^25–27^.

Therefore, efforts need to be made to develop this understanding level to promote CVDs awareness, prevent, control, and successfully manage cardiovascular disease among the Syrian general community, so far we suggested many proposals to resolve this severe issue as follows:

- enhancing public health promotion via mass media campaigns such as television, radio, and billboard commercials, as well as text messaging educating the public on the warning signals of heart attacks, stroke, and CVD risk factors, are possible tools for improving CVD knowledge and overall cardiovascular health of communities, especially Especially among young people^28^.
- Adopting these health promotion efforts may be improved by including community health professionals in their execution. This is significant, particularly for low socioeconomic groups where we found relatively little information yet the highest illness burden.
- Other objectives for consideration by the Syrian ministry of public health include the early beginning of such awareness programs through school-based interventions (incorporating them into the curriculum), teaching youngsters about heart health and risk factors, and other effective activities elsewhere^29^.
- Overall, there is an urgent need for funding to conduct interventional and longitudinal studies to assess the efficacy of these measures on future CVD burden. Importantly, in order to continue identifying concerns that need attention, these efforts and strategies should be implemented in tandem with ongoing monitoring and assessment of their adoption as well as an examination of trends in CVD.
- Programs to modify stress-related behaviours and pay greater attention to at-risk populations are crucial.
- Improving socioeconomic circumstances is one of the most important steps that must be taken in order to reduce the incidence of cardiovascular diseases via the correction of risk factors, namely stress and behaviour variables.
- Promoting healthy lifestyles includes exercising regularly, quitting smoking, maintaining a low cholesterol level, maintaining a clean diet, and maintaining optimum body weight.
- Using safe, effective medications to manage cardiovascular disease and increasing their access to low-income communities.
- Encouragement and engagement in global and international activities via education, volunteering, and research participation to promote the discovery of solutions based on shared information.
- Since armed conflict significantly contributes to the development of chronic illnesses, including CVDs, efforts must be made to discover ways to end armed conflict.

### Limitations and strength

Our research is a cross-sectional study of the current kind, which may not provide the actual generality findings at the level of the studied population as good associations between the findings and the exposure couldn't be detected due to they are examined at the same time, even though it is a free and simple way of data collection. It is hard to eliminate bias since the survey was sent through a Google Form to social networking platforms, making it impossible for the elderly, those living in rural areas, and illiterate people who do not have access to the internet or do not have email, to take the survey. Even though we listed a lot of limitations, there are also a lot of advantages, such as the fact that we distributed the questions around and collected answers from all Syrian governments, as well as the fact that we used both closed-ended and open-ended questions to prevent bias in the responses. It was difficult to measure participants' height and weight since our study was based on a questionnaire, and the determination of weight and height were measured depending on the participant's confidence in their responses. Since the survey provided answers about height and weight in the form of categories rather than actual numbers, we were unable to determine the respondent's body mass index. The questionnaire mainly included yes/no questions regarding smoking and alcohol consumption, with no further details about frequency or quantity. Since this is an online cross sectional study, it is difficult to represent actual awareness levels of CVDs and generalize the finding on the population. A professional investigator was tracing the data collection process in order to limit the possibility bias, repeated answers, and illogical responses.

## Conclusion

Our results demonstrate a great gap in knowledge about cardiovascular diseases between open and closed questions. A paradigmatic change is required to harness the capacity of healthcare professionals as conduits of health information to enhance public awareness of risk factors and warning signals, particularly in rural regions and among youngsters. Because of the terrible scenario that exists in Syria at present in all respects, especially the inadequate knowledge of common severe diseases such as CVDs, both international and local healthcare organizations should be conducting many beneficial courses and awareness programmers that explain everything about CVDs, especially how we may avoid the predominance of these illnesses.

## Supplementary Information

Supplementary Table S1.

## Data Availability

The authors have access to and have saved all of the data necessary to support this paper's conclusion. All data are accessible upon reasonable request from the first author.
