# Publisher Correction: Syrians' awareness of cardiovascular disease risk factors and warning indicators: a descriptive cross-sectional study

**DOI:** 10.1038/s41598-023-35321-2

**Published:** 2023-05-24

**Authors:** Sarya Swed, Hidar Alibrahim, Haidara Bohsas, Wael Hafez, Mohammed Amir Rais, Sheikh Shoib, Ebraheem Albazee, Mohamed E. G. Elsayed, Bisher Sawaf, Amr Farwati, Mohammed Najdat Seijari, Naim Battikh, Nour Shaheen, Nafeth Ibrahem, Ahmad Alsaleh, Ka Yiu Lee, Amine Rakab

**Affiliations:** 1 Faculty of Medicine, Aleppo University, Aleppo, Syria; 2 NMC Royal Hospital, 16th Street, Khalifa City, Abu Dhabi, UAE; 3 Medical Research Division, Department of Internal Medicine, The National Research Centre, Cairo, Egypt; 4 Faculty of Medicine of Algiers, University of Algiers, Algiers, Algeria; 5 JLNM Hospital, Rainawari, Srinagar, India; 6 Directorate of Health Services, Srinagar, J&K India; 7 Kuwait Institute for Medical Specializations, Kuwait City, Kuwait; 8 Department of Psychiatry and Psychotherapy III, University of Ulm, Leimgrubenweg 12-14, 89075 Ulm, Germany; 9 Department of Psychiatry, School of Medicine and Health Sciences, Carl von Ossietzky University Oldenburg, Oldenburg, Germany; 10 Department of Internal Medicine, Hamad Medical Corporation, Doha, Qatar; 11 John H. Stroger, Jr. Hospital of Cook County, Chicago, USA; 12 Faculty of Medicine, Alexandria University, Alexandria, Egypt; 13 Faculty of Medicine, Damascus University, Damascus, Syria; 14 Department of Health Sciences, Mid Sweden University, Sundsvall, Sweden; 15 Weill Cornell Medical College, Doha, Qatar

Correction to: *Scientific Reports* 10.1038/s41598-023-32026-4, published online 25 April 2023

The original version of this Article contained errors. *P* values in Table 4 were dislocated.

The original Table 4 appears below.

**Table 4:** The Difference between the sub-groups of knowledge toward risk factors, and warning signs of CVDs among the study sample.

Variable	Categories	Risk factors			Warning signs			Risk factors and warning signs		
		Mean (SD) 8.47 (0.077)	95%CI (lower–upper)	*P* value	Mean (SD) 5.81 (0.097)	95%CI (lower–upper)	*P* value	Mean (SD) 14.28 (0.153)	95%CI (lower–upper)	*P* value
Age group		< 0.001		< 0.001		< 0.001				
	18–24	8.35 (0.07)	8.22–8.49		5.7 (0.083)	5.53–5.86		14.05 (0.136)	13.78–14.32	
	25–34	8.36 (0.138)	8.09–8.63		5.55 (0.180)	5.2–5.91		13.91 (0.271)	13.38–14.45	
	35–44	8.49 (0.148)	8.2–8.78		5.87 (0.213)	5.45–6.29		14.36 (0.299)	13.77–14.95	
	45–54	9 (0.166)	8.67–9.33		6.75 (0.202)	6.35–7.15		15.75 (0.314)	15.12–16.37	
	55–64	9.15 (0.16)	8.83–9.47		6.38 (0.258)	5.87–6.9		15.54 (0.356)	14.83–16.25	
Gender		0.071		0.445		0.205				
	Female	8.44 (0.064)	8.31–8.56		5.82 (0.078)	5.66–5.97		14.25 (0.125)	14.01–14.5	
	Male	8.51 (0.091)	8.33–8.68		5.81 (0.116)	5.58–6.04		14.32 (0.181)	13.96–14.67	
Place of residency		0.183		0.011		0.016				
	City	8.51 (0.061)	8.39–8.63		5.9 (0.077)	5.75–6.05		14.41 (0.121)	14.17–14.65	
	Countryside	8.34 (0.106)	8.13–8.55		5.57 (0.129)	5.32–5.83		13.91 (0.202)	13.51–14.31	
Level of education		< 0.001		0.013		< 0.001				
	No formal education	8.61 (0.371)	7.84–9.38		5.83 (0.53)	4.71–6.94		14.43 (0.659)	13.07–15.8	
	Primary school	6.55 (0.743)	4.89–8.2		5 (0.831)	3.15–6.85		11.55 (1.442)	8.33–14.76	
	Middle school	7.71 (0.377)	6.95–8.48		5.12 (0.409)	4.29–5.95		12.83 (0.639)	11.54–14.12	
	Secondary school	7.66 (0.165)	7.34–7.99		5.42 (0.166)	5.09–5.75		13.08 (0.284)	12.52–13.64	
	University (Bachelor's or Master's)	8.63 (0.055)	8.52–8.74		5.91 (0.73)	5.77–6.05		14.54 (0.113)	14.32–14.77	
Marital status		0.095		0.050		0.026				
	Single	8.42 (0.063)	8.29–8.54		5.69 (0.078)	5.53–5.84		14.1 (0.124)	13.86–13.35	
	Married	8.52 (0.103)	8.32–8.73		6.1 (0.128)	5.85–6.35		14.62 (0.202)	14.23–15.02	
	Divorced	8.85 (0.335)	8.15–9.55		6.7 (0.524)	5.6–7.8		15.55 (0.709)	14.07–17.03	
	Widowed	9.13 (0.427)	8.2–10.04		5.69 (4.33)	4.33–7.05		14.81 (0.853)	13.0–16.63	
Monthly income		0.089		0.900		0.521				
	High	8.61 (0.378)	7.84–9.37		5.95 (0.468)	5–6.9		14.55 (0.744)	13.05–16.06	
	Good	8.55 (0.09)	8.37–8.72		5.85 (0.111)	5.64–6.07		14.4 (0.177)	14.05–14.75	
	Middle	8.39 (0.07)	8.26–8.53		5.8 (0.087)	5.63–5.97		14.19 (0.137)	13.92–14.46	
	Low	8.55 (0.19)	8.18–8.93		5.72 (0.259)	5.21–6.23		14.27 (0.385)	13.51–15.04	
Occupation		< 0.001		< 0.001		< 0.001				
	Student	8.36 (0.077)	8.21–8.52		5.58 (0.094)	5.4–5.77		13.95 (0.151)	13.65–14.24	
	Farmer	8.41 (0.429)	7.5–9.32		5.65 (0.717)	4.13–7.17		14.06 (0.941)	12.06–16.05	
	House wife	7.69 (0.238)	7.22–8		5.44 (0.225)	5–5.89		13.14 (0.416)	12.31–13.96	
	Employed	8.51 (0.134)	8.25–8.78		5.78 (0.175)	5.43–6.12		14.29 (0.262)	13.77–14.81	
	Petty business	8.98 (0.2)	8.58–9.38		6.35 (0.315)	5.72–4.98		15.34 (0.448)	14.44–16.23	
	Medical sector	9.05 (0.11)	8.84–9.27		6.7 (0.145)	6.42–6.99		15.76 (0.224)	15.31–16.2	
	Other	8.1 (0.188)	7.73–84.7		5.44 (0.245)	4.96–5.93		13.55 (0.364)	12.82–14.27	

The original Article has been corrected.

