# Supplementary material for: Syrians' awareness of cardiovascular disease risk factors and warning indicators: a descriptive cross-sectional study
Source: Sci Rep. 2023 Apr 25;13:6764. doi: 10.1038/s41598-023-32026-4 (PMC10130054; doi:10.1038/s41598-023-32026-4)
Supplement: Supplementary file 1 — Supplementary Table S1. [file 41598_2023_32026_MOESM1_ESM.docx]

**Socio-demographic Data:**

- Age: *
- Gender: *
- Male
- Female
- Original Residence: *
- Rural
- Urban
- Educational level: *
- N/A
- Primary
- Preparatory
- Secondary
- University
- Postgraduate
- College: *
- Unmarried
- Married
- Divorced
- Widower
- Separate
- Economic level: *
- Bad
- Middle
- Good
- Excellent
- Do you belong to the medical sector? *
- YES
- NO
- Which of the following best describes your main job or occupation over the past year? (Please add the profession in the other field if it is not present in the options)? *
- Farmer
- Doctor
- House wife
- employee/ worker
- Business/Commerce
- Student
- Retired
- Unemployed
- Pharmacist
- Others:
- How much is your tall? *
- less than 150
- 150 _ 159
- 160 _ 169
- 170 _ 179
- 180 _ 189
- More than 190
- How much is your tall? *
- less than 50
- 50 _ 59
- 60 _ 69
- 70 _ 79
- 80 _ 89
- More than 90
- Do you smoke? *
- YES
- NO
- Do you drink alcohol? *
- YES
- NO
- Do you have a history of diabetes? *
- YES
- NO
- Do you suffer from high blood pressure? *
- YES
- NO
- Do you suffer from high blood pressure? *
- YES
- NO
- Does anyone in your family have a heart disease? *
- YES
- NO
- Does anyone in your family have high blood pressure? *
- YES
- NO
- Does anyone in your family have diabetes? *
- YES
- NO
- Are you concerned about having a heart disease? *
- YES
- NO
- Are you concerned about high blood pressure? *
- YES
- NO
- Are you concerned about getting diabetes? *
- YES
- NO

*Knowledge about risk factors for CVDs*

1. Do you know the risk factors for cardiovascular diseases?

- YES
- NO

| 1. *If yes, can you mention the risk factors for cardiovascular diseases?*   *(LET THE PARTICIPANT MENTION, AND CIRCLE ALL THAT APPY)* | *Advanced/Older age*  *Overweight/Obesity*  *hypertension*  *Diabetes mellitus*  *Dyslipidemia*  *Smoking*  *Excessive alcohol consumption*  *Physical inactivity*  *Family history*  *Stress*  *Don’t know* | *= 1*  *= 2*  *= 3*  *= 4*  *= 5*  *= 6*  *= 7*  *= 8*  *= 9*  *= 10*  *= 88* |
| --- | --- | --- |

| 1. How many cardiovascular disease risk factors have been mentioned by participant?   *DK = 88* | Number | └─┴─┘ |
| --- | --- | --- |

| Which of the following are risk factors for cardiovascular diseases?  *(READ OUT TO THE CLIENT AND RECORD FOR EACH)* | | | |
| --- | --- | --- | --- |
| Older age | Yes = 1 No = 2 | DK = 88 |  |
| Obesity/Overweight | Yes = 1 No = 2 | DK = 88 |  |
| Hypertension | Yes = 1 No = 2 | DK = 88 |  |
| Diabetes mellitus | Yes = 1 No = 2 | DK = 88 |  |
| Dyslipidemia | Yes = 1 No = 2 | DK = 88 |  |
| Smoking | Yes = 1 No = 2 | DK = 88 |  |
| Excessive alcohol consumption | Yes = 1 No = 2 | DK = 88 |  |
| Physical inactivity | Yes = 1 No = 2 | DK = 88 |  |
| History of heart disease | Yes = 1 No = 2 | DK = 88 |  |
| Family history | Yes = 1 No = 2 | DK = 88 |  |
| Stress | Yes = 1 No = 2 | DK = 88 |  |
| Kidney disease | Yes = 1 No = 2 | DK = 88 |  |

*Knowledge about warning signs for cardiovascular disease*

| Do you know the warning signs for cardiovascular disease event | Yes = 1 | No = 2 |
| --- | --- | --- |
| If yes, can you mention the warning signs for cardiovascular disease event?  (LET THE PARTICIPANT MENTION, AND CIRCLE ALL THAT APPY) | Headache  Chest pain  Dyspnea  Sweating  Vomiting  Pain in the teeth/jaw  Pain/numbness in the arm  Loss of consciousness  Dizziness or light headedness  Don’t know | = 1  = 2  = 3  = 4  = 5  = 6  = 7  = 8  = 9  = 88 |

| How many warning signs have been mentioned by the participant?  *DK = 88* | Number | └─┴─┘ |
| --- | --- | --- |

| Which of the following are warning signs for cardiovascular diseases event?  *(READ OUT TO THE CLIENT AND RECORD FOR EACH)* | | | |
| --- | --- | --- | --- |
| Headache | Yes = 1 No = 2 | DK = 88 |  |
| Chest pain | Yes = 1 No = 2 | DK = 88 |  |
| Dyspnea | Yes = 1 No = 2 | DK = 88 |  |
| Sweating | Yes = 1 No = 2 | DK = 88 |  |
| Vomiting | Yes = 1 No = 2 | DK = 88 |  |
| Pain in the teeth or jaw | Yes = 1 No = 2 | DK = 88 |  |
| Pain or numbness in the arm | Yes = 1 No = 2 | DK = 88 |  |
| Loss of consciousness | Yes = 1 No = 2 | DK = 88 |  |
| Dizziness or light headedness | Yes = 1 No = 2 | DK = 88 |  |

| Which of the following foods are good/healthy for prevention of cardiovascular diseases  *(READ OUT TO THE CLIENT AND RECORD FOR EACH)* | | | |
| --- | --- | --- | --- |
| Green leafy vegetables | Yes = 1 No = 2 | DK = 88 |  |
| Fruits | Yes = 1 No = 2 | DK = 88 |  |
| Salty foods | Yes = 1 No = 2 | DK = 88 |  |
| Fast foods | Yes = 1 No = 2 | DK = 88 |  |
| Whole grain foods | Yes = 1 No = 2 | DK = 88 |  |
| High fat diet | Yes = 1 No = 2 | DK = 88 |  |
